# Cold-inducible RNA-binding protein causes endothelial dysfunction via activation of Nlrp3 inflammasome

**DOI:** 10.1038/srep26571

**Published:** 2016-05-24

**Authors:** Weng-Lang Yang, Archna Sharma, Zhimin Wang, Zhigang Li, Jie Fan, Ping Wang

**Affiliations:** 1 Center for Immunology and Inflammation, The Feinstein Institute for Medical Research, Manhasset, NY, USA; 2 Department of Surgery, Hofstra Northwell School of Medicine, Manhasset, NY, USA; 3 Department of Surgery, University of Pittsburgh School of Medicine, Pittsburgh, PA, USA

## Abstract

Cold-inducible RNA-binding protein (CIRP) is a damage-associated molecular pattern (DAMP) molecule which stimulates proinflammatory cytokine release in hemorrhage and sepsis. Under these medical conditions, disruption of endothelial homeostasis and barrier integrity, typically induced by proinflammatory cytokines, is an important factor contributing to morbidity and mortality. However, the role of CIRP in causing endothelial dysfunction has not been investigated. In this study, we show that intravenous injection of recombinant murine CIRP (rmCIRP) in C57BL/6 mice causes lung injury, evidenced by vascular leakage, edema, increased leukocyte infiltration and cytokine production in the lung tissue. The CIRP-induced lung damage is accompanied with endothelial cell (EC) activation marked by upregulation of cell-surface adhesion molecules E-selectin and ICAM-1. Using *in vitro* primary mouse lung vascular ECs (MLVECs), we demonstrate that rmCIRP treatment directly increases the ICAM-1 protein expression and activates NAD(P)H oxidase in MLVECs. Importantly, CIRP stimulates the assembly and activation of Nlrp3 inflammasome in MLVECs accompanied with caspase-1 activation, IL-1β release and induction of proinflammatory cell death pyroptosis. Finally, our study demonstrates CIRP-induced EC pyroptosis in the lungs of C57BL/6 mice for the first time. Taken together, the released CIRP in shock can directly activate ECs and induce EC pyroptosis to cause lung injury.

In sepsis, trauma and hemorrhagic shock, proinflammatory cytokines and damage-associated molecular pattern molecules (DAMPs) released from cells of injured tissue have been shown to activate polymorphonuclear cells (PMNs) and endothelial cells (ECs), leading to systemic inflammation and multiple-organ dysfunction^1^,^2^,^3^,^4^,^5^. The activated EC layer becomes permeable and loses the integrity of EC barrier, which allows fluids, pathogens and excessive leukocytes entering into tissues to cause damage^6^. The formation of inflammasome is another cellular response to pathogens or DAMPs for proinflammatory cytokine release^7^. The inflammasome is a multimolecular platform which assembles as a complex of nucleotide-binding oligomerization domain-like receptor (Nlr) proteins, apoptosis-associated speck-like protein containing a caspase activation and recruitment domain (ASC), and caspase-1 (or caspase-5) to release proinflammatory cytokines IL-1β and IL-18^7^. The Nlr family, pyrin domain containing 3 (Nlrp3) inflammasome can assemble in response to a wide variety of stimuli and has been implicated in triggering inflammation in a wide variety of disease pathologies, making it the most versatile and clinically implicated inflammasome^7^. The Nlrp3 inflammasome activation typically occurs in phagocytes^7^ but recent studies have also shown it in ECs^8^,^9^,^10^,^11^,^12^. Moreover, activation of caspase-1 via inflammasome can trigger pyroptosis, an inflammatory form of programmed cell death subroutine that mostly occurs in macrophages^13^, but has not yet demonstrated in ECs.

Cold-inducible RNA-binding protein (CIRP, official gene name Cirbp) is a highly conserved 172-amino acid nuclear protein that belongs to the family of cold shock proteins^14^. CIRP contains one amino-terminal consensus sequence RNA-binding domain and one carboxy-terminal glycine-rich domain^15^. CIRP functions as an RNA chaperone facilitating mRNA translation^16^,^17^ and is ubiquitously expressed in various tissues at low levels^15^,^18^,^19^. CIRP is induced upon mild hypothermia^14^,^19^,^20^, by exposure to ultraviolet irradiation^21^,^22^, and hypoxia^23^. It has also been described as a new generation proto-oncogene^24^. Recently, we discovered that CIRP is a new inflammatory mediator or DAMP^25^. During shock, CIRP is released into the circulation and stimulates cytokine release in macrophages^25^,^26^,^27^,^28^. However, whether the released CIRP has any other role in contributing to the endothelial damage has not been well explored.

In this study, we hypothesized that the released CIRP directly contributes to the vascular EC injury through the Nlrp3 inflammasome pathway. We first conducted an *in vivo* study by injecting mice with recombinant murine CIRP (rmCIRP) and examined for vascular leakage, tissue edema, neutrophil infiltration and expression of proinflammatory cytokines TNF-α and IL-1β as well as cell-surface adhesion molecules E-selectin and ICAM-1 as markers for EC activation in the lungs. To determine whether the EC activation in the lung tissue is a direct effect in response to rmCIRP administration, we treated isolated primary mouse lung vascular ECs (MLVECs) with rmCIRP and examined the expression of EC activation marker. We further determined the mechanism for CIRP-induced EC activation by examining NAD(P)H oxidase activation and formation of Nlrp3 inflammasome in rmCIRP-treated MLVECs. Finally, we identified that rmCIRP induced EC pyroptosis *in vitro* and *in vivo*.

## Results

### Extracellular CIRP causes vascular leakage and endothelial cell activation in the lungs

**Figure 1:**
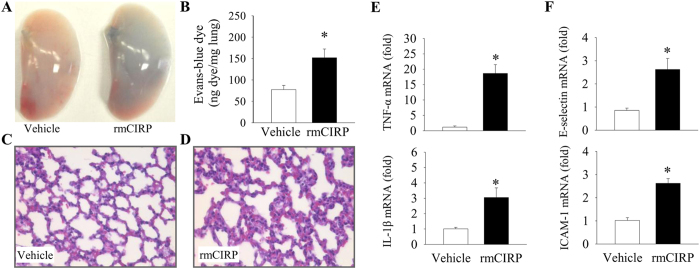
Extracellular CIRP induces vascular leakage and causes lung injury. The C57BL/6 mice were intravenously injected with vehicle (normal saline) or recombinant murine CIRP (rmCIRP, 5 mg/kg body weight) and lungs were collected for various analysis 5 h later. **(A)** Representative images of the lungs stained with Evans blue dye (EBD) post-vehicle or rmCIRP injections. **(B)** Colorimetric analysis of EBD extracted from stained lungs. Bars represent mean ± SEM (n = 5 per group). *P < 0.05; Student’s *t*-test. Representative images of H&E stained lung sections from **(C)** vehicle or **(D)** rmCIRP injected mice at original magnification ×400. The qPCR analysis of **(E)** cytokines and **(F)** endothelial cell adhesion molecules in the lungs of vehicle or rmCIRP injected mice. Bars represent mean ± SEM (n = 3 per group). *P < 0.05; Student’s t-test.

The lungs are the most susceptible organ for injury in hemorrhagic or septic shock^29^. CIRP acts as an inflammatory mediator in hemorrhagic and septic shock^25^. In our previous study, we have demonstrated that intravenous (i.v.) administration of 5 mg/kg body weight rmCIRP to healthy mice increases the levels of serum proinflammatory cytokines (TNF-α, IL-6 and HMGB1) and liver enzymes (AST and ALT)^25^. To examine the role of CIRP in contributing to the lung injury, we i.v. injected healthy mice with the same dose of rmCIRP to study its effects on the vascular integrity in the lungs. Evans blue dye (EBD) assay is commonly used to assess blood vessel permeability *in vivo*. When ECs become dysfunctional and lose their close contacts, the i.v. injected EBD leaks into the tissues^30^. At 5 h after rmCIRP injection, the lungs of mice had increased permeability shown as more blue coloration than the lungs of vehicle-injected mice (Fig. 1A). The amount of the EBD extravasated in the lungs of rmCIRP-injected mice was 2-fold higher than that in the vehicle-injected mice (Fig. 1B).

The pulmonary parenchyma of mice injected with rmCIRP also had significant edema and neutrophil infiltration in comparison with that of vehicle group mice (Fig. 1C,D), as judged by H&E staining. In addition, rmCIRP increased the lung expression of proinflammatory cytokines TNF-α and IL-1β mRNA by 16.3- and 3.0- fold, respectively, compared to the vehicle at 5 h after injection (Fig. 1E). These results indicate that CIRP causes an increase in vascular permeability, neutrophil infiltration and inflammation for the lung jury. We next examined the markers for activated status of ECs in the lungs at 5 h after rmCIRP injection by measuring gene expression of the EC adhesion molecules using qPCR. The mRNA levels of E-selectin and intercellular adhesion molecule (ICAM)-1 in the lungs of mice injected with rmCIRP was 3.1- and 2.6- fold higher than those injected with vehicle, respectively (Fig. 1F). Upregulation of EC adhesion molecules after rmCIRP injection indicate that extracellular CIRP can cause activation of ECs in the lung tissue.

### CIRP directly activates lung endothelial cells

**Figure 2:**
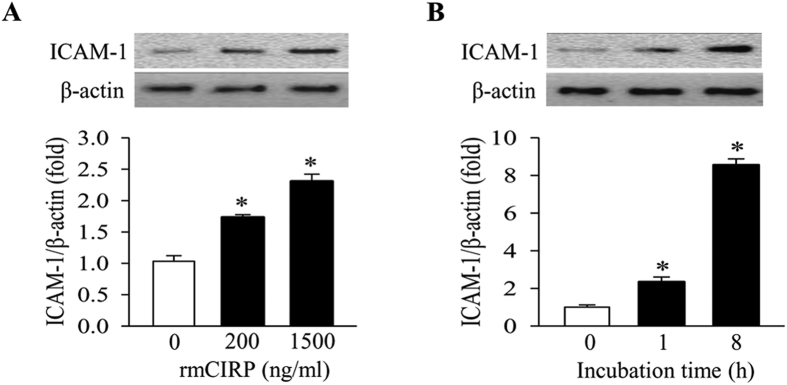
CIRP activates vascular endothelial cells. **(A)** Western blot analysis of ICAM-1 in the mouse lung vascular endothelial cells (MLVECs) treated with an indicated concentration of rmCIRP for 4 h. The image shown is representative of three independent experiments. Bars represent mean ± SEM (n = 3) from densitometric analysis of blots. *P < 0.05 compared to no rmCIRP; one-way ANOVA, Student-Newman-Keuls test. **(B)** Western blot analysis of ICAM-1 in MLVECs treated with 200 ng/ml rmCIRP for upto 8 h as indicated. The image is representative of three independent experiments. Bars represent mean ± SEM (n = 3). *P < 0.05 compared to time 0; one-way ANOVA, Student-Newman-Keuls test.

To determine whether CIRP can have a direct effect on ECs, we isolated primary mouse lung vascular endothelial cells (MLVECs) and then treated them with rmCIRP. The induction of ICAM-1 is a well-known indicator of EC activation. The addition of rmCIRP induced the expression of ICAM-1 in MLVECs in a dose-dependent manner, increasing it by 1.7-fold at 200 ng/ml and by 2.3-fold at 1,500 ng/ml, compared to the absence of rmCIRP at 4 h (Fig. 2A). We then used 200 ng/ml of rmCIRP to conduct a time-course study of the EC activation by rmCIRP. The ICAM-1 levels in MLVECs increased by 2.3-fold at 1 h and further increased by 8.5-fold after 8 h of rmCIRP treatment (Fig. 2B). Taken together, these results indicate that CIRP specifically activates ECs in dose- and time-dependent manners.

### CIRP stimulates NAD(P)H oxidase in lung endothelial cells

**Figure 3:**
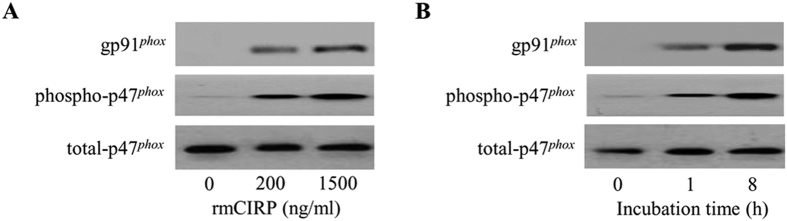
CIRP induces NAD(P)H oxidase activation. Western blot analysis of gp91^*phox*^, phosphorylated p47^*phox*^ (phospho-p47^*phox*^) and total p47^*phox*^ from mouse lung vascular endothelial cells (MLVECs) immunoprecipitated with anti-p47^*phox*^ antibody after treatment with **(A)** an indicated concentration of rmCIRP for 4 h or **(B)** 200 ng/ml rmCIRP for upto 8 h as indicated. The images are representative of three independent experiments.

Reactive oxygen species (ROS) generated from NAD(P)H oxidase play an important role in activating ECs during lung inflammation, ischemia/reperfusion injury, sepsis, hyperoxia, and ventilator-associated lung injury^31^. NAD(P)H oxidase is a complex of multimeric enzymes consisting of 5 subunits, gp91^*phox*^ (*phox* for phagocytic oxidase), p22^*phox*^, p47^*phox*^, p67^*phox*^, and small GTPase Rac^31^. Upon stimulation, the p47^*phox*^ subunit becomes phosphorylated and binds to gp91^*phox*^, generating a conformational change of the entire complex and resulting in its activation^31^. After treatment of MLVECs with rmCIRP, we observed an increase of the binding of gp91^*phox*^ to phosphorylated p47^*phox*^ in a dose-dependent manner by immunoprecipitation with anti-p47^*phox*^ antibody (Fig. 3A). We also observed that the binding of gp91^*phox*^ to phosphorylatedp47^*phox*^ was increased from 1 h to 8 h in the MLVECs treated with 200 ng/ml of rmCIRP (Fig. 3B). These data show that CIRP activates NAD(P)H oxidase and suggest a critical role of endogenous NAD(P)H oxidase in mediating CIRP-induced EC activation.

### CIRP induces the formation of Nlrp3 inflammasome in lung endothelial cells

**Figure 4:**
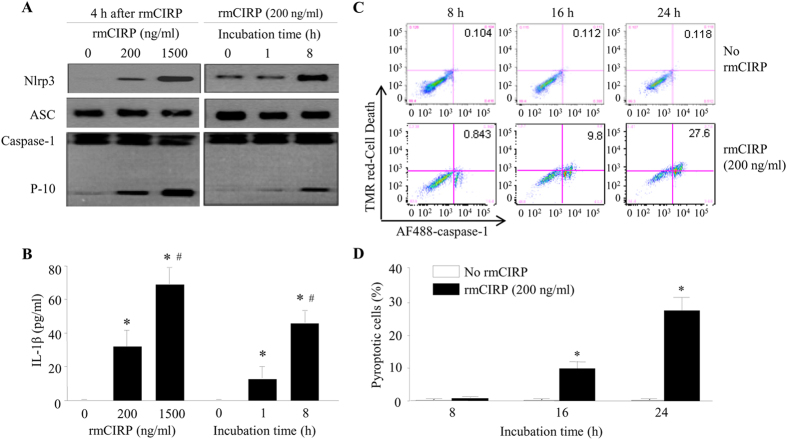
CIRP induces Nlrp3 inflammasome activation and pyroptosis *in vitro*. **(A**,**B)** Mouse lung vascular endothelial cells (MLVECs) were treated with an indicated concentration of rmCIRP for an indicated period of time. **(A)** The cells from the treatments were subjected to immunoprecipitation with anti-ASC antibody. Immunoblot analysis of Nlrp3, ASC and cleaved caspase-1 was performed. The images are representative of three independent experiments. **(B)** IL-1β levels in the cell culture media from above treatments, measured by ELISA. Bars represent mean ± SEM (n = 3). *P < 0.05 compared to no rmCIRP or 0 h; ^#^*P* < 0.05 compared to 200 ng/ml rmCIRP or 1 h; one-way ANOVA, Student-Newman-Keuls test. **(C**,**D)** MLVECs were treated with no rmCIRP or 200 ng/ml rmCIRP for upto 24 h as indicated. Cells were then stained with TMR-red-Cell Death reagent and Alexa Fluor 488-FLICA-activated caspase-1 fluorescent reagent (AF488-caspase-1), followed by flow cytometry. **(C)** Representative dot blots showing percentages of double-stained pyroptotic MLVECs as indicated by numbers in the top right quadrant. **(D)** Graph showing the percentages of pyroptotic cells from flow cytometric analysis. Bars represent mean ± SEM (n = 3). *P < 0.05 compared to no rmCIRP; one-way ANOVA, Student-Newman-Keuls test.

The NAD(P)H oxidase-derived ROS has been shown to be a contributing factor for the formation of Nlrp3 inflammasome in ECs^8^. We then assessed CIRP’s ability to assemble and activate Nlrp3 inflammasome to release IL-1β. A major component of the inflammasome is ASC, an adaptor protein that mediates the interaction between Nlrp3 and caspase-1^7^. To test whether CIRP induced the inflammasome in ECs, we treated MLVECs with rmCIRP at different doses and for different times. We observed an increase of the interaction between Nlrp3 and ASC in a dose-dependent manner by immunoprecipitation with anti-ASC antibody (Fig. 4A). We also observed that the association of Nlrp3 and ASC in MLVECs was induced at 1 h after incubation with 200 ng/ml rmCIRP and was further increased by 8 h (Fig. 4A). Another characteristic of inflammasome formation is the activation of caspase-1, which can be detected by its cleaved P-10 subunit^7^. In the same setting as for examining the formation of Nlrp3-ASC complex, we also detected an increase of P-10 levels in MLVECs treated with rmCIRP in dose- and time- dependent manners (Fig. 4A). Accordingly, rmCIRP also induced release of IL-1β by MLVECs in a dose-dependent manner, with 32.4-fold increase at 200 ng/ml and 69.5-fold increase at 1,500 ng/ml (Fig. 4B). Furthermore, IL-1β levels were elevated by 11.8-fold at 1 h and by 42.4-fold at 8 h after rmCIRP addition (Fig. 4B). Altogether, these data demonstrate a NLRP3 inflammasome-mediated mechanism of IL-1β release and EC activation by CIRP.

### CIRP induces lung endothelial cell pyroptosis *in vitro* and *in vivo*

**Figure 5:**
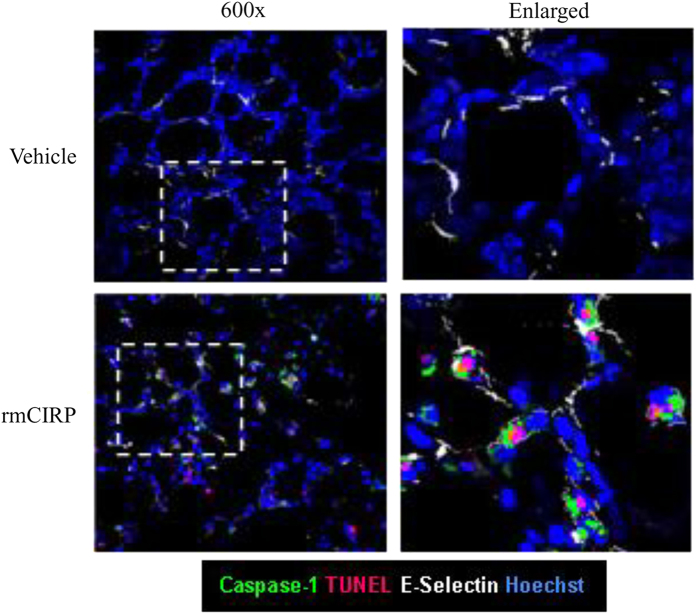
CIRP induces endothelial cell pyroptosis *in vivo*. C57BL/6 mice were intravenously injected with vehicle (normal saline) or rmCIRP (5 mg/kg body weight). After 24 h, lung tissues were harvested and subjected to fluorescent immunohistochemistry analysis. Representative images of lung tissues viewed under confocal microscopy (original magnification x600) after staining with Alexa Fluor 488- FLICA-activated caspase-1 (green), TUNEL (red), APC-E-selectin-1 (white) and Hoechst (blue). Images are representative of three independent experiments.

We next examined whether the activation of EC inflammasome by CIRP could lead to pyroptosis. By staining the cells with Cell Death and caspase-1 activity reagents and analyzing with flow cytometry, we detected significant increase in the percentage of pyroptotic MLVECs after rmCIRP (200 ng/ml) treatment, which progressed in a time-dependent manner (Fig. 4C,D). The percentage of pyroptotic MLVECs was negligible without rmCIRP treatment (Fig. 4C,D), whereas 8 h after addition of rmCIRP 0.8% MLVECs were pyroptotic which increased by 12-fold (9.8%) after 16 h and by 35-fold (28%) after 24 h of rmCIRP treatment (Fig. 4D). To validate the observation *in vivo*, we i.v. injected healthy mice with rmCIRP and 24 h later harvested and subjected the lung tissues to immunofluorescent analysis. Lung ECs stained with E-selectin (white fluorescence) exhibited positive staining for activated caspase-1 (green fluorescence) and DNA fragmentation (red fluorescence as assessed by TUNEL) in the rmCIRP-treated mouse, while it was not observed in the vehicle-treated control mouse (Fig. 5). Taken together, our *in vitro* and *in vivo* studies clearly indicate that CIRP can directly induce pyroptosis in ECs.

## Discussion

We report here that extracellular CIRP, which is an endogenous DAMP, causes endothelial dysfunction in a direct manner and induces lung damage. We show that administration of CIRP to mice results in vascular leakage, edema, increased leukocyte infiltration and proinflammatory cytokines TNF-α and IL-1β in the lung tissues along with upregulation of surface adhesion molecules E-selectin and ICAM-1, reflecting EC activation. CIRP induces EC activation in a direct manner by demonstrating in cultured MLVECs. The NAD(P)H oxidase in ECs is also activated in the presence of CIRP. Importantly, our findings demonstrate that CIRP activates the Nlrp3 inflammasome in ECs, resulting in IL-1β release and caspase-1 mediated induction of EC pyroptosis.

Pulmonary vascular dysfunction and injury are characterized by disruption of the normal microvascular alveolo-capillary permeability barrier, resulting in extra-vascular leak of protein-rich edema into the pulmonary interstitial and alveolar compartments as well as increased PMN sequestration/adhesion in the lungs^32^,^33^. Our results clearly show that intravenous administration of rmCIRP to healthy mice resulted in the development of lung edema, increased leukocyte accumulation and increased production of TNF-α and IL-1β in the lungs. In this study, we have further shown that rmCIRP administration increases the expression of E-selectin and ICAM-1 as well as induces vascular leakage in the lungs of mice. Endothelial dysfunction has been linked to EC activation which is defined by the endothelial expression of cell-surface adhesion molecules, such as E-selectin and ICAM-1^34^. Other DAMPs such as HMGB1, mitochondrial DNA, S100 family proteins and Shiga toxin have also been shown to cause endothelial dysfunction and change endothelial permeability^35^,^36^,^37^,^38^.

The ICAM-1 expressed in ECs is a counter receptor for the leukocyte β_2_-integrins LFA-1 and Mac-1 and plays an important role in the regulation of PMN sequestration^39^. The interaction of ICAM-1 with these integrins enables the PMN to adhere firmly to the vascular endothelium and migrate across the microvascular barrier^39^. EC activation is known to be typically induced by proinflammatory cytokines such as TNF-α, IFN-γ and IL-6^40^. We have previously shown that CIRP treatment results in increased TNF-α release from cultured macrophages and CIRP administration *in vivo* results in increased serum levels of TNF-α and IL-6^25^. Through induction of cytokines, CIRP can indirectly induce EC activation *in vivo.* We have further examined the possibility of direct EC activation by CIRP and shown that treatment with rmCIRP increases the ICAM-1 expression and the IL1-β release in cultured MLVECs in a dose- and time- dependent manner. Thus, the released CIRP in hemorrhagic and septic shock can both directly and indirectly cause endothelial activation and dysfunction, leading to the lung damage.

Reactive oxygen species (ROS) function as important signaling molecules to mediate various biological responses. Excessive production of ROS results into oxidative stress and contributes to various pathophysiological responses including lung injury^31^. Phagocytes such as neutrophils and monocytes are best known to generate large quantities of ROS using the enzyme NAD(P)H oxidase^41^. The nonphagocytic ECs also contain NAD(P)H oxidase, which is the major enzymatic mechanism to generate ROS within ECs^42^. The neutrophil NAD(P)H oxidase-derived ROS have been shown to be an important regulator of EC activation^43^ for the inflammatory responses after trauma and hemorrhage. In this study, we demonstrate that CIRP is directly capable of inducing the NAD(P)H oxidase activation in ECs. NAD(P)H oxidases are a group of multimeric enzymes, which consist of cytosol complex including p40^*phox*^, p47^*phox*^, and p67^*phox*^ and membrane components p22^*phox*^ and gp91^*phox*^. Upon stimulation, p47^*phox*^ is phosphorylated and the entire cytosolic complex migrates to the membrane where it associates with gp91^*phox*^ and cytochrome b_558_ to assemble the active oxidase^31^. Since CIRP did not alter total p47^*phox*^ expression, as shown in Fig. 3, CIRP seems to be a potent stimulator of NAD(P)H oxidase activation by promoting enzymes assembly, rather than by upregulating the expression of the enzyme molecules. Recently, NAD(P)H oxidase has been shown to be required for Nlrp3 inflammasome activation and IL-1β release in ECs in response to hemorrhage^8^, in macrophages in pulmonary fibrosis^44^, and in podocytes in response to hyperhomocyteinemia^45^ and diabetic nephropathy^46^. In the same token, we also observe that NAD(P)H oxidase activation by CIRP is associated with the formation of Nlrp3 inflammasome in ECs.

Dysregulated Nlrp3 inflammasome activation is associated with both heritable inflammatory diseases such as Muckle-Wells syndrome, familial cold autoinflammatory syndrome and neonatal onset multi-system inflammatory disease as well as acquired inflammatory diseases such as Alzheimer’s disease, gout, pseudogout, obesity, atherosclerosis, metabolic syndrome, type 2 diabetes mellitus and age-related macular degeneration^47^. A growing list of many endogenous DAMPs, such as extracellular ATP, uric acid crystals, cholesterol crystals, hyaluronan, heparan sulfate, calcium pyrophosphate dihydrate, β-amyloid plaques and islet amyloid polypeptide, also induce Nlrp3 inflammasome activation after their accumulation or alteration under damage or disease conditions^47^. Although Nlrp3 inflammasome activation was originally thought to be immune cell specific, it has recently been studied in non-immune cells such as ECs during hemorrhage^8^,^12^, atherosclerosis^10^, coronary arteritis^9^ and obesity^11^. Our results show that CIRP induced the assembly of Nlrp3 inflammasome in ECs resulting in the activation of caspase-1 and IL-1β production, providing another proinflammatory activity of CIRP besides activating macrophages for cytokine release^25^. Inflammasome activation is initiated through the assembly of the existing, but not *de novo* synthesis of, components. This is also supported by our observation that CIRP did not change the protein expression of ASC and pro-caspase-1, as shown in Fig. 4A.

Cell death critically influences the inflammatory process^48^,^49^. The death of ECs under pathologic conditions can be through a variety of different cell death mechanisms such as apoptosis, necrosis, necroptosis, and autophagy. Proinflammatory cytokines such as IFN-γ have been shown to exacerbate endothelial injury by triggering EC death via caspase-8-dependent apoptosis^50^. In another study, pulmonary microvascular dysfunction has been shown to be due to MLVEC death via caspase-dependent apoptosis mediated by NAD(P)H oxidase signaling in a murine sepsis model^51^. Pyroptosis is a newly characterized inflammasome-mediated caspase-1-dependent cell death subroutine that mostly occurs in macrophages^13^,^52^. Pyroptosis is morphologically and mechanistically distinct from apoptosis and necrosis as it features plasma membrane rupture and release of inflammatory factors^52^. It occurs in response to a number of bacterial and viral pathogens but can also be triggered by stroke, heart attack, hemorrhage, or cancer^13^. Recently, HMGB1 was reported to induce inflammasome activation and pyroptosis in macrophages^53^. HMGB1 has been shown to trigger Nrlp3 inflammasome-mediated caspase-1 activation in ECs in hemorrhagic shock and uraemia^8^,^54^, but EC pyroptosis has not been demonstrated in these studies. We show that extracellular CIRP causes EC pryoptosis both *in vitro* and *in vivo*. Our study reports a novel mechanism by which the CIRP, an endogeneous DAMP, can mediate activation of Nlrp3 inflammasome and caspase-1 as well as induces EC pyroptosis, further enhancing the EC dysfunction. Whether like HMGB1, CIRP can also induce Nlrp3 inflammasome activation and pyroptosis in macrophages as well as other cell types needs further investigation.

**Figure 6:**
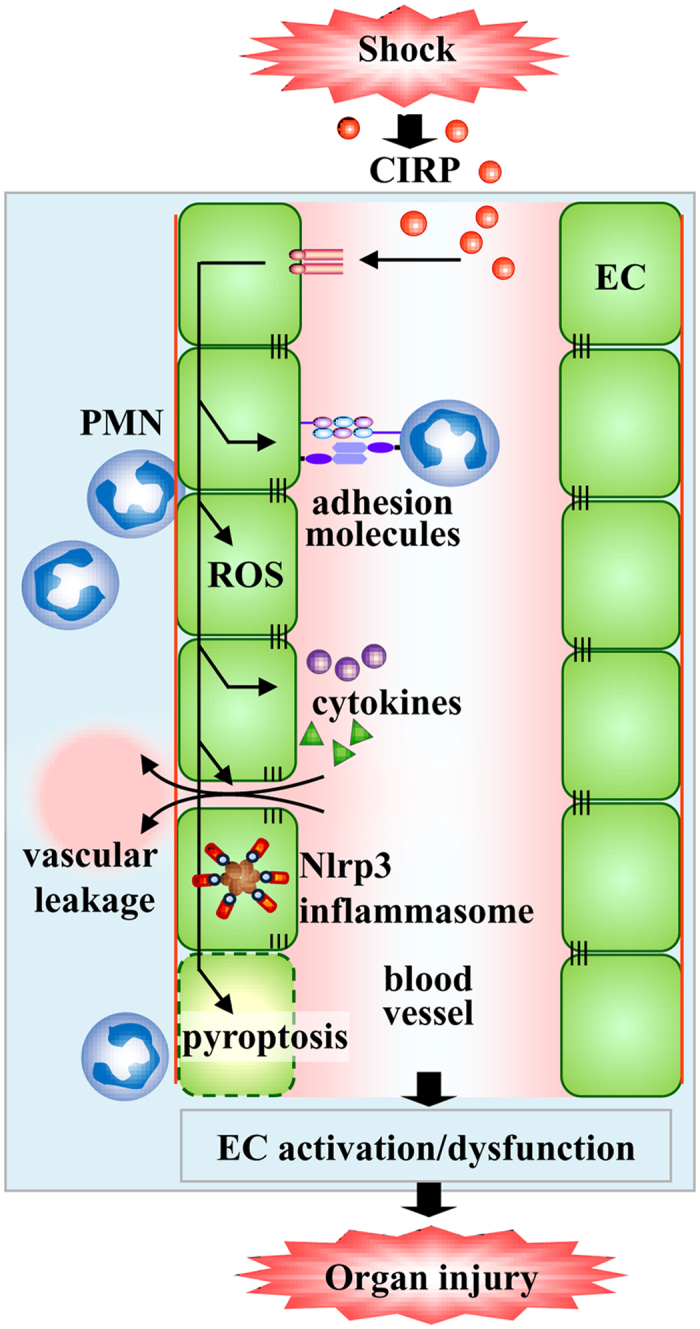
Model of CIRP-induced vascular endothelial cell dysfunction. CIRP released during hemorrhagic or septic shock activates endothelial cells (EC) by upregulating adhesion molecules on them to increase infiltration of polymorphonuclear leukocytes (PMN) and produce proinflammatory cytokines and reactive oxygen species (ROS). CIRP also induces activation of Nlrp3 inflammasome and caspase-1-mediated EC pyroptosis. This series of events serve as a mechanism underlying the CIRP-induced EC dysfunction which then leads to organ injury.

The pathogenesis of vascular EC dysfunction is complex^55^. Based on this study, we propose a mechanistic model for CIRP-induced vascular EC dysfunction leading to organ injury (Fig. 6). During hemorrhagic and septic shock, CIRP is released in circulation and in turn activates ECs which is defined by the upregulation of adhesion molecules and production of proinflammatory cytokines to increase PMN infiltration. CIRP also induces NAD(P)H oxidase-mediated generation of ROS and Nlrp3 inflammasome activation in ECs leading to caspase-1-mediated EC pyroptosis. This study suggests that targeting CIRP as an adjunct therapy for acute inflammatory diseases via attenuation of EC injury could be an important translational approach.

## Methods

### Mice

Male C57BL/6 mice (20 to 25 g) purchased from the Jackson Laboratory (Bar Harbor, ME, USA) were used in all experiments. These mice were housed in a temperature-controlled room on a 12 hour light/dark cycle in the animal facility within the Feinstein Institute for Medical Research or University of Pittsburgh and fed a standard laboratory diet. All experiments were performed in accordance with the recommendations in the Guide for the Care and Use of Laboratory Animals of the National Institutes of Health (Bethesda, MD, USA). All the animal experimental protocols were reviewed and approved by the Institutional Animal Care and Use Committees (IACUC) of the Feinstein Institute for Medical Research, University of Pittsburgh and VA Pittsburgh Healthcare System. All efforts were made to minimize suffering.

### Administration of rmCIRP

rmCIRP was produced as described previously^25^. Mice were allocated to two groups: vehicle and rmCIRP. A small incision on the neck was made and the internal jugular vein was exposed. Normal saline (vehicle) or rmCIRP at a dose of 5 mg/kg body weight (BW) in 200 μl volume was delivered by injection using 29G × 1/2″ U-100 insulin syringe (Terumo Medical Corporation, Elkton, MD, USA) through the jugular vein. At 5 h after rmCIRP injection, mice were anesthetized and lungs were collected. A section of lung tissue was preserved in 10% formalin for histopathological analysis and rest was frozen in liquid nitrogen and stored at −80 °C for qPCR analysis. An additional set of experiments were performed with Evans blue injected at 5 h after rmCIRP injection and the lungs were harvested for evaluation.

### Lung permeability assay

The changes in vascular permeability after rmCIRP administration were measured by Evans blue dye (EBD) leakage from blood into lung airways^30^. EBD (20 mg/kg; Sigma-Aldrich, St. Louis, MO) in 100 μl volume was administered intravenous (i.v.) by tail vein injection 1 h before the end of the 5 h experiments. One hour later, lungs were perfused with normal saline through the spontaneously beating right ventricle to remove intravascular dye. Lungs were removed, documented by photographs, and dried at 60 °C for 48 h. Evans blue dye was extracted from lungs by incubating in formamide (Sigma-Aldrich) at 37 °C for 24 h and centrifuged at 5,000*g* for 30 min. The EBD in the supernatant was quantitated by a dual wavelength (620 and 740 nm) spectrophotometric method, correcting for contaminating heme pigments by using the formula OD_620_ (EBD) = OD_620_ − (1.426 × OD_740_ + 0.030)^56^. The extravasated EBD concentration in lung homogenate was calculated against a standard curve and expressed as the dye incorporated per mg of tissue.

### Histologic examination

The lung tissues were fixed in 10% formalin followed by paraffin embedding. The paraffin tissue blocks were cut into 5 μm sections, which were transferred to glass slides and stained with hematoxylin and eosin (H&E). Morphologic changes in the lung tissues were examined by light microscopy, documented by photographs and evaluated by two investigators in a blinded manner. Lung injury was assessed based on pulmonary edema judged by thickened alveolar walls with vascular congestion and interstitial and alveolar leukocyte infiltration.

### RNA extraction and quantitative real-time PCR

Total RNA was isolated from lung tissue by TRIzol reagent (Invitrogen, Carlsbad, CA, USA) following the manufacturer’s instruction and was reverse-transcribed into cDNA using murine leukemia virus reverse transcriptase (Applied Biosystems, Foster City, CA, USA). A PCR reaction was carried out in a 24 μl final volume containing 0.08 μM of each forward and reverse primer, cDNA, and 12 μl SYBR Green PCR Master Mix (Life Technologies, Grand Island, NY). The following gene-specific primers were used for amplifying genes: E-selectin forward, 5′-TGACATCGTCCTCATTGCTC-3′, and reverse, 5′-TTTCCTGCTGTCTTCAGCTTATC-3′; ICAM-1 forward, 5′-GGGCTGGCATTGTTCTCTAA-3′, and reverse, 5′-CTTCAGAGGCAGGAAACAGG-3′; TNF-α forward, 5′-AGACCCTCACACTCAGATCATCTTC-3′, and reverse, 5′-TTG CTACGACGTGGGCTACA-3′; IL-1β forward, 5′-CAGGATGAGGACATGAGCACC -3′, and reverse, 5′-CTCTGCAGACTCAAACTCCAC -3′; and β-actin forward, 5′-CGTGAAAAGATGACCCAGATCA -3′ and reverse, 5′-TGGTACGACCAGAGGCATACAG -3′. Amplification was conducted in an Applied Biosystems 7300 real-time PCR machine (Applied Biosystems) under the thermal profile of 50 °C for 2 min and 95 °C for 10 min, followed by 40 cycles of 95 °C for 15 s and 60 °C for 1 min. The data was analyzed by the 2^−ΔΔCt^ method for relative quantization and normalized to mouse β-actin mRNA. Relative expression of mRNA was expressed as the fold change in comparison with the vehicle injected lung tissue.

### Mouse lung vascular EC isolation and culture

Mouse lung vascular ECs (MLVEC) were isolated using a previously described method^57^ with modifications^8^. Briefly, mice were anesthetized by intraperitoneal administration of 50 mg/kg ketamine and 5 mg/kg xylazine and the chest cavity was opened. The right ventricle was cannulated to infuse PBS for removing blood from lungs. Peripheral lung tissue was diced to ∼1 mm^3^ pieces and cultured in a 60-mm culture dish in growth medium (MEM D-Val medium [Invitrogen Gibco] containing 2 mM glutamine, 10% FBS, 5% human serum, 50 μg/ml penicillin/streptomycin, 5 μg/ml heparin, 1 μg/ml hydrocortisone, 80 μg/ml endothelial cell growth supplement from bovine brain, 5 μg/ml amphotericin, and 5 μg/ml mycoplasma removal agent) at 37 °C with 5% CO_2_ for 60 h. The tissue dices were removed and the adherent cells were continued in culture for 3 d. The MLVECs were purified from these cultured cells using biotin-conjugated rat anti-mouse CD31 (PECAM-1) mAb and BD IMag streptavidin particles plus-DM, and the immunomagnetic separation system (BD Biosciences, San Jose, CA, USA) following the manufacturer’s instructions. The purified MLVECs were allowed to grow for 3 to 4 d and then characterized by their cobblestone morphology, uptake of Dil-Ac-LDL (Biomedical Technologies, Stoughton, MA, USA), and staining for factor VIII-related Ag (Sigma-Aldrich). MLVECs from 3 to 5 passages were used in experiments in which cells were treated with increasing concentrations of rmCIRP (200 and 1500 ng/ml) for 4 h or treated with 200 ng/ml rmCIRP for various time points, washed with HBSS three times, and harvested for further analysis.

### Immunoblot analysis and coimmunoprecipitation

MLVECs were lysed (∼1 × 10^6^ cells/ml) in lysis buffer (10 mM Tris [pH 7.4], 150 mM NaCl, 5 mM EDTA, 1% Triton X-100, 10 mM NaF, 1 mM Na_3_VO_4_, 10 μg/ml leupeptin, 10 μg/ml aprotinin, and 20 mM PMSF). Protein concentrations were determined by Bio-Rad Laboratories (Hercules, CA, USA) protein assay reagent. 30 μg total protein for each sample was fractionated on a 10% SDS-PAGE gel and then transfered onto polyvinylidene difluoride (PVDF) membrane. The membranes were then blocked for 1 h at room temperature with blocking buffer (LI-COR Biosciences, Lincoln, NE, USA) and probed with anti-ICAM-1 or anti-β-actin antibodies (Santa Cruz Biotechnologies, Santa Cruz, CA, USA). The membranes were then washed with PBS for three times and incubated with the appropriate secondary antibody (LI-COR Biosciences). Protein bands were detected using LI-COR Odyssey Fc Imager (LI-COR Biosciences). Blots were quantitated using NIH Image J densitometric software and normalized to β-actin. For coimmunoprecipitation studies, 600 μg total protein for each sample was immunoprecipitated with anti-p47^*phox*^ or anti-ASC antibodies (Santa Cruz Biotechnologies). The immunoprecipitated proteins were then fractionated on 10% SDS-PAGE gel and immunoblotted as described above with anti-phosphoserine antibody (Invitrogen) or anti-gp91^*phox*^ or anti-NLRP3 antibodies (Santa Cruz Biotechnologies) and detected with Clean-Blot IP Detection Reagent (Thermo Scientific, Rockford, IL, USA) following the manufacturer’s instructions. Blots were then stripped and reprobed with anti-p47^*phox*^ or anti-ASC or anti-mouse caspase-1 p10 antibodies (Santa Cruz Biotechnologies).

### Flow cytometry analysis of cell pyroptosis

MLVECs were stimulated with 200 ng/ml rmCIRP or left untreated for 8–24 h followed by staining of the cells with Alexa Fluor 488-labeled Fluorochrome Inhibitor of Caspase-1(FLICA) (ImmunoChemistry Technologies, Bloomington, MN, USA), which binds to activated caspase-1, at 37  °C for 1 h. After fixing with 4% paraformaldehyde, cells were stained with tetramethylrhodamine (TMR) red-labeled *In-Situ* Cell Death Detection reagent (Roche Applied Science, Indianapolis, IN, USA) following the manufacturer’s instructions. The cells were then analyzed by flow cytometry. Background and auto-fluorescence were determined by a control antibody with the same isotype staining. Acquisition was performed on 10,000 events using a FACScalibur cytometer (BD Biosciences) or BD LSR II (BD Biosciences) and the data was analyzed by CellQuestPro (BD Biosciences) and FlowJo-V10 software (Tree Star, Ashland, OR, USA). The percentage of double-stained cells was taken as percentage of pyroptotic cells.

### Measurement of IL-1β

IL-1β in cell-culture media was measured using the ELISA Ready-Set-Go kit for mouse IL-1β (eBioscience, San Diego, CA, USA) following the manufacturer’s instructions.

### Immunofluorescence confocal microscopy

Lung tissues were harvested from vehicle (normal saline) and rmCIRP (5 mg/kg body weight) injected mice after 24 h of i.v. injections and subjected to multi-fluorescent immunohistochemistry analysis. Briefly, lung sections were fixed and stained with 1× cell permeable Alexa Fluor 488-labeled FLICA (ImmunoChemistry), followed by TUNEL staining (Roche Applied Science) for dead cells, nuclear staining with 1 μg/ml Hoechst 33258 (Sigma-Aldrich) and allophycocyanin (APC)-labeled E-selectin-1 (R&D Systems, Minneapolis, MN) staining for ECs. The stained lung section was imaged by confocal microscope with excitation at 480 nm for green fluorescence of caspase-1–positive cells, at 540 nm for red fluorescence of TUNEL-stained cells, at 365 nm for blue fluorescence of Hoechst-stained nuclei and at 650 nm for white fluorescence of E-selectin positive ECs.

### Statistical analysis

Data were expressed as mean ± standard error of the mean (SEM) and analyzed using SigmaPlot11 graphing and statistical analysis software (Systat Software Inc., San Jose, CA, USA). Student’s *t* test was used for comparing two groups. Multiple groups were compared by one-way analysis of variance (ANOVA) with Student-Newman-Keuls’ (SNK) test. Differences were considered significant if *P* < 0.05.

## Additional Information

**How to cite this article**: Yang, W.-L. *et al*. Cold-inducible RNA-binding protein causes endothelial dysfunction via activation of Nlrp3 inflammasome. *Sci. Rep.* **6**, 26571; doi: 10.1038/srep26571 (2016).
